# A pilot study of a nurse-delivered cognitive behavioral therapy intervention (Ziphamandla) for adherence and depression in HIV in South Africa

**DOI:** 10.1177/1359105316643375

**Published:** 2016-04-26

**Authors:** Lena S Andersen, Jessica F Magidson, Conall O’Cleirigh, Jessica E Remmert, Ashraf Kagee, Matthew Leaver, Dan J Stein, Steven A Safren, John Joska

**Affiliations:** 1 University of Cape Town, South Africa; 2 Massachusetts General Hospital/ Harvard Medical School, USA; 3 Fenway Health, USA; 4 Stellenbosch University, South Africa; 5 Medical Research Council Unit on Anxiety and Stress Disorders, South Africa; 6 University of Miami, USA

**Keywords:** adherence, cognitive behavioral therapy, depression, HIV, low- and middle-income countries, South Africa, task-sharing

## Abstract

Depression is prevalent among people living with HIV in South Africa and interferes with adherence to antiretroviral therapy. This study evaluated a nurse-delivered, cognitive behavioral therapy intervention for adherence and depression among antiretroviral therapy users with depression in South Africa (*n* = 14). Primary outcomes were depression, antiretroviral therapy adherence, feasibility, and acceptability. Findings support robust improvements in mood through a 3-month follow up. Antiretroviral therapy adherence was maintained during the intervention period. Participant retention supports acceptability; however, modest provider fidelity despite intensive supervision warrants additional attention to feasibility. Future effectiveness research is needed to evaluate this nurse-delivered cognitive behavioral therapy intervention for adherence and depression in this context.

## Introduction

Depression is prevalent among people living with HIV (PLWH). In South Africa, where the greatest number of PLWH reside worldwide, rates of depression have been found to be 41 percent among PLWH (Freeman et al., 2008). Depression is associated with compromised immune functioning and accelerated disease progression among PLWH (Leserman et al., 1999) and sub-optimal antiretroviral therapy (ART) adherence (Gonzalez et al., 2011; Nakimuli-Mpungu et al., 2012; Uthman et al., 2014). ART non-adherence in South Africa, and other low- and middle-income countries (LMICs), has serious consequences as it may lead to drug resistance in a setting where limited ART regimens are available.

Addressing depression in HIV care in South Africa has potential to improve HIV treatment and prevention; however, it is estimated that only one in four patients with a psychiatric disorder receive appropriate treatment (Seedat et al., 2008). This is in part due to lack of access to specialized mental health providers in South Africa. Psychologists and psychiatrists are in short supply with 0.28 psychiatrists and 0.32 psychologists per 100,000 population working in the public health sector (Lund et al., 2010). Although psychotropic medication is typically available in primary care, resources for evidence-based psychotherapeutic interventions are scarce (Lund et al., 2010). In a resource-constrained context, such as South Africa, task-sharing, whereby non-mental health specialists provide mental health services under the supervision of specialists, may be the most feasible approach for delivering mental health services in primary care (Saraceno et al., 2007).

An effective and feasible therapy model for depression that could be integrated into primary HIV care in South Africa is needed. Cognitive behavioral therapy (CBT) is a therapy model with a strong empirical base for the treatment of depression. Research on the effectiveness of CBT for depression in PLWH has shown promising results (Safren et al., 2009, 2012; Spies et al., 2013). In the United States, an intervention that integrates CBT for depression with a cognitive behavioral approach to adherence counseling (cognitive behavioral therapy intervention for adherence and depression (CBT-AD)) has been found to be effective in improving adherence and reducing depression in PLWH and on the United States–Mexico border with Mexican patients (Safren et al., 2004, 2009, 2012; Simoni et al., 2013). Furthermore, there is prior evidence that CBT can be delivered by non-mental health specialists (Malik et al., 2009; Nicholas, 2014; Rahman et al., 2008), including in sub-Saharan Africa (Bere et al., 2018).

Based on previous quantitative (Andersen, 2009) and qualitative studies (Andersen et al., 2015) conducted by our research team in peri-urban areas outside Cape Town, we developed an intervention based on CBT-AD for delivery using a task-sharing model (i.e. with clinical nurse practitioners) called Ziphamandla (Empower Yourself). This pilot study was a first step to adapting and examining the applicability and usefulness of Ziphamandla in treating adherence and depression in ART users in South Africa. The aims of this study were to assess if Ziphamandla was (1) feasible and acceptable to patients and providers (measured by patient retention and provider fidelity to intervention delivery) and (2) associated with improvements in depressive symptoms, functioning, and ART adherence.

## Method

### Setting

The study was conducted in Khayelitsha, one of the largest peri-urban areas in the Western Cape (~400,000 people), where approximately 20 percent of people are HIV-positive (Strategic Development Information and GIS Department, 2011). Recruitment and treatment took place at two of the busiest primary HIV care clinics in the area, referred to here as Site 1 and Site 2.

### Study design and procedures

#### Adaptation of CBT-AD

A South African team consisting of clinical psychologists, psychiatrists, and a psychiatric nurse, with input and support from the US team who developed CBT-AD, planned for study design and the adaptation of CBT-AD to the South Africa context (Safren et al., 2009, 2012). The three main considerations were (1) feasibility of rolling out the intervention, (2) the cultural relevance and comprehensibility of the treatment content, and (3) the training schedule and materials for the interventionists.

#### Feasibility

To ensure feasibility of roll-out, two important decisions were made with regard to study design: (1) location of treatment delivery needed to be primary HIV clinics to ensure accessibility for patients in Khayelitsha and (2) the chosen cadre of interventionist needed to represent staff present at the clinics (i.e. clinical nurse practitioners). Furthermore, three major decisions were made to accommodate delivery in primary health care using a task-sharing model: first, the number of treatment sessions was reduced to a maximum of eight and included a range of six to eight sessions to allow for exploration of optimal session length. Second, the cognitive restructuring module was removed. The team ultimately decided that the time needed to train and supervise non-specialists in this technique reduced long-term feasibility of rolling out this intervention in primary care, which was a primary consideration. Prior research has found behavioral activation to be as effective as the full CBT package including cognitive restructuring in the treatment of depression (Jacobson et al., 2000), thus the team felt confident that the treatment protocol would be as valuable without the inclusion of the cognitive restructuring module.

Third, the decision was made to tailor the order of the treatment modules to the individual needs of each patient. Each patient’s particular situation dictated the order in which the problem-solving, behavioral activation and relaxation modules were administered following the Life Steps adherence intervention session.

#### Training schedule and materials

The team recognized the importance of an adapted, structured, easy-to-use manual for intervention delivery, with a checklist of session content for the interventionists to use as a guide throughout each session.

#### Cultural relevance/comprehensibility of the treatment content

The terminology used in the manual was modified to make it more user-friendly to non-mental health specialists. Examples used in the original intervention were culture-specific to the United States and were replaced with items culturally relevant to South Africa. For example, items such as “snow shoeing” were removed from the activity list and replaced with “hair braiding.” All worksheets and patient forms were translated into isiXhosa. Specific cultural adaptations and modifications are listed in Appendix 1.

#### Training and supervision

Training occurred once a week for 4 hours. After 88 hours of training, the interventionists underwent an evaluation to determine their readiness to begin administering treatment. This was assessed by a written test of the treatment content and a role-play of a random session. The interventionists were required to score above 80 percent on the written test to be deemed ready to begin administering treatment. Supervision occurred once a week in two separate 2-hour sessions. The morning sessions were spent reviewing and giving feedback on therapy sessions that had taken place throughout the week and setting goals for the upcoming therapy sessions. The afternoon sessions were spent reviewing the treatment content for the next therapy sessions (i.e. providing ongoing training on the intervention).

#### Participants

Eligibility criteria for this study consisted of being an adult (18+ years), being fluent in either isiXhosa or English, meeting diagnostic criteria for major depressive disorder (MDD) as assessed by the Mini International Neuropsychiatric Interview 6.0 (MINI), being HIV-positive, and using ART. Moreover, it was a requirement that the participants had obtained knowledge of their HIV-positive status at least 6 months prior to the study assessment date to ensure appropriate MDD diagnosis. Participants were excluded from the study if they were actively suicidal or psychotic or if they had an uncontrolled neurological problem. Additional exclusion criteria included (1) having been initiated on or had their dose of psychotropic medication altered within the past 3 months, (2) currently receiving psychotherapy for depression, or (3) having previously received CBT. None of the patients who met criteria for MDD on the MINI met these exclusion criteria. A total of 14 participants, 7 from each of the two sites, were recruited for the study from September 2011 to June 2012.

#### Recruitment

A recruitment nurse conducted screenings of patients in the designated HIV treatment waiting areas at Sites 1 and 2. Patients were identified based on the day’s list of patients waiting to receive their ART medication refills. Clinic staff had been briefed on the study and they referred patients to the study. At Site 2, a screening program, which had been implemented by a non-governmental organization (Breuer et al., 2012, 2014), was also used for referrals. The research assistant screened patients who consented to participate using the Center for Epidemiological Studies Depression Scale (CES-D). Patients who screened positive (CES-D ⩾20) were scheduled for an assessment interview with the study psychologist and the research assistant.

#### Procedure

Individuals who met study eligibility criteria and were interested in participating in the study provided informed consent and then a baseline assessment was conducted (measures listed below). The first Ziphamandla treatment session was scheduled for the following week at their clinic. All treatment sessions were audio recorded with the consent of the participants. Assessments of self-reported depressive symptoms and objective ART adherence were conducted weekly prior to the start of each therapy session. Clinician assessments were re-administered at the end of treatment and at 3-month follow up. The study was approved by the University of Cape Town Human Research Ethics Committee.

### Self-report assessments

Self-report assessments were administered in isiXhosa by a bilingual study research assistant and included the following.

#### CES-D

The CES-D is a 20-item self-report questionnaire of depressive symptoms, including sadness, sleep disturbance, and loss of appetite. Each item is scored from 0 to 3. There are four positive items that are reverse coded for calculating the total score. The CES-D has been shown to have excellent validity and reliability in previous research in South Africa (Pretorius, 1991), and the scale has been used in studies of PLWH in South Africa (Myer et al., 2008). When the CES-D was validated against the MINI in a study of PLWH in South Africa, a cut-off of 20 in detecting MDD yielded a sensitivity of 79 percent and specificity of 61 percent (Myer et al., 2008).

#### Sheehan Disability Scale

The 3-item Sheehan Disability Scale (SDS) assesses impairment in three domains (work, family life, and social life). The three domains are rated on a Likert scale from 0 to 10, with higher scores indicating more impairment. The SDS has demonstrated good reliability and validity in assessing psychiatric impairment including impairment caused by depression (Leon et al., 1997). It has previously been used in studies conducted among PLWH in South Africa (Andersen, 2009; Olley et al., 2004).

The CES-D and SDS were translated using Brislin’s (1970) classic back-translation model. Back-translation is a well-known method used to maintain equivalence when translating source language to target language (Behling & Law, 2000). The measures were translated and back translated into isiXhosa, checked by a third bilingual translator, and then administered in isiXhosa.

### Clinician-administered assessments

A study psychologist administered the clinician assessments in English with the support of the research assistant for translation into isiXhosa when necessary. These included the following.

#### MINI

Diagnostic evaluations were conducted using the Mini International Diagnostic Interview 6.0 (MINI). The MINI is a structured diagnostic interview that elicits all the symptom criteria specified in the *Diagnostic and Statistical Manual of Mental Disorders* (4th ed.; DSM-IV) and International Classification of Diseases–10th Revision (ICD-10) for 19 Axis I and II disorders and suicidality (Sheehan et al., 1998). It was created for use in international clinical settings and research. The MINI demonstrates good reliability and validity. It has been validated against the Structured Clinical Interview for DSM diagnosis (SCID-P) and the Composite International Diagnostic Interview for ICD-10 (Sheehan et al., 1998).

#### Hamilton Depression Scale

The Hamilton Depression Scale (HAM-D) is a 17-item clinician-administered assessment of depression severity (Hamilton, 1959). Each item is scored from 0 to 4, with the total score being an aggregate of all item scores. Higher scores indicate greater severity. The HAM-D is the most widely used measure of treatment outcome in MDD studies and has been shown to have satisfactory psychometric properties in the general population (Bagby et al., 2004). The HAM-D has been used in prior trials in South Africa, with a cut-off of ⩽6 indicating depression remission (e.g. Kennedy and Emsley, 2006).

### ARV adherence

Adherence was monitored during treatment with real-time medication monitoring, (i.e. Wisepill technology; Haberer et al., 2010). Wisepill uses Internet and cell-phone-based technology to allow investigators to monitor in real time when a pillbox, containing participants’ ART medication, has been opened. Adherence scores were calculated by dividing the number of times the pillbox was opened during each week by the number of doses prescribed for the week. A dose was considered missed if it was not taken within the 2-hour window of the prearranged dose time. A 2-week baseline period was established to obtain adherence data for each participant before the intervention was administered. In addition to determining weekly adherence scores, the percentage of the sample that maintained ART adherence ⩾80 percent throughout the study period was assessed given recent evidence of the clinical importance of treatment interruptions for viral suppression (Parienti et al., 2008).

### Therapist fidelity rating procedure

Upon completion of all treatment sessions, one session from each participant and seven sessions by each nurse were randomly selected and translated into English by a bilingual translator (14 of the 90 session recordings; 15.6%). Two study psychologists independently rated fidelity and quality of the treatment administration using two rating scales drawn from the original CBT-AD studies (Safren et al., 2009, 2012). The one rating scale focuses on nine generic CBT strategies, which should occur in every session. These include collaboratively setting the agenda, reviewing medication adherence, and assigning homework. A Likert scale (0 to 6) is used to score each item. The other rating scale focuses on the session-specific content. For example, in the first behavioral activation session, items such as “Goes through the positive events checklist” were rated from 0 to 2 to indicate did not complete (0), completed but not satisfactorily (1), and completed satisfactorily (2). Fidelity ratings were recorded as a percentage of components completed for each session (two separate percentages for generic CBT strategies and session-specific content).

### Treatment attendance

Therapy sessions were scheduled weekly. The length of treatment was tailored to patient needs (ranging from six to eight sessions). Based on participants’ progress, the study nurses and psychologists decided on the number of sessions each participant warranted. Treatment completion was defined as greater than five treatment sessions attended.

### Overview of statistical analyses

Generalized linear mixed models (GLMM; GENLINMIXED) in SPSS (version 21) were used for all longitudinal analyses. Change in clinician-rated depressive symptoms (HAM-D) was assessed from baseline to the 3-month follow up (i.e. three time points). Change in self-reported depressive symptoms (CES-D) was assessed from baseline to the 3-month follow up and weekly at each therapy session (up to 10 assessments total). Wisepill adherence assessments were only available from baseline to post-treatment (weekly data at each therapy session; up to eight assessments total). GLMM has an advantage in which it can accommodate dependent variables that are non-normally distributed and can accommodate missing data. For each analysis, the appropriate model was selected based on the distribution of the main outcome variable (e.g. linear or gamma for positively skewed outcomes). In all models, the main effect of time was entered as a fixed effect. Due to the small sample size, no random effects or intercepts were included in the model. Degrees of freedom were determined via the Satterthwaite approximation, which is preferred over the residual method when sample size is small, as in this study. The autoregressive (AR-1) covariance structure was used to account for the distinct time intervals of assessments. All models employed robust estimation (which handles violations of model assumptions). Finally, as a metric of effect size, Cohen’s *d* was computed for each outcome within subjects from baseline to the last assessment time point (3-month follow up for all outcomes with the exception of Wisepill adherence), accounting for the correlation between means at the two time points.

## Results

### Participant demographics

All participants identified their ethnic origin as Xhosa (the second largest tribal group in South Africa). In all, 13 of the participants were female and 1 was male. Participants ranged in age from 28 to 59 years. See Table 1 for demographic information.

**Table 1.** Demographic information of all participants (*n* = 14).

Variable	Mean (SD)
Age (years)	38.4 (7.81)
Highest grade level	9.1 (2.9)
Monthly income	1115.7 (595.9) ZAR
% (*n*)	
Sex	
Female	92.9 (13)
Home language	
isiXhosa	100 (14)
Race/ethnicity	
Xhosa	100 (14)
Work situation	
Unemployed	85.7 (12)
Marital status	
Single	57.1 (8)
Common law	7.1 (1)
Widowed	7.1 (1)
Separated	14.3 (2)
Type of housing	
Shack	64.3 (9)
House	28.6 (4)
Running water	57.1 (8)
Electricity	92.9 (13)
Cell phone	92.9 (13)

SD: standard deviation.

### Treatment attendance

In all, 12 of the 14 patients completed the Ziphamandla treatment (i.e. >5 treatment sessions). Three-month follow-up data were obtained from 11 of the 12 participants who completed treatment and from one of the two participants who discontinued treatment.

### Therapist fidelity

Across both raters for all sessions reviewed, a mean of 54 percent of generic CBT elements were addressed and 63 percent of session-specific components (percentages are the proportion of the intervention content that was addressed for both general CBT elements and session-specific components). Agreement between raters was assessed based on each rating that was within 10 percent of each other for the two raters (examining the session-specific content and more general CBT ratings separately). For the generic CBT ratings, the raters were in agreement for 11 out of 14 sessions (78.6%). For the session-specific ratings, the raters were in agreement for 9 out of the 14 sessions (64.3%).

### CES-D

A generalized mixed model was tested with CES-D from baseline to the 3-month follow up (i.e. 3 months after completion of treatment) as the main outcome (including weekly CES-D assessments; up to 10 time points total). Self-reported depression scores significantly decreased over time (*γ* = −0.18, standard error (SE) = 0.03, 95% confidence interval (CI) = [−0.25, −0.11], *t*(41) = −6.08, *p* < .0001), from baseline (*M* = 40.9, standard deviation (SD) = 8.45) to the 3-month follow up (*M* = 6.58, SD = 7.61), with a very large effect size (Cohen’s *d* = 3.8).

### HAM-D

A generalized mixed model was tested with HAM-D from baseline to the 3-month follow up as the main outcome (three time points total). Clinician-rated depression scores significantly decreased over time (*γ* = −0.71, SE = 0.18, 95% CI [−1.15, −0.28], *t*(17) = −3.89, *p* < .01) from baseline (*M* = 26.4, SD = 5.5) to the 3-month follow up (*M* = 5.8, SD = 5.8), with a very large effect size (Cohen’s *d* = 2.5).

### Sheehan disability

A generalized mixed model was tested examining Sheehan scores at baseline to the 3-month follow up as the main outcome (three time points total). There was a significant decrease in functional impairment over time (*γ* = −35.6, SE = 3.35, 95% CI [−42.49, −28.63], *t*(21) = −10.60, *p* < .0001) from baseline (*M* = 76.50, SD = 22.17) to the 3-month follow up (*M* = 6.08, SD = 14.29), with a very large effect size (Cohen’s *d* = 2.94).

### Wisepill adherence

Wisepill adherence was captured from baseline to post-treatment (up to 8 time points total). Adherence remained stable over the course of the study period (*γ* = 0.03, SE = 0.07, 95% CI [−0.11, 0.17], *t*(76) = −0.45, *p* = .65; Cohen’s *d* = 0.1). Of those who completed treatment (*n* = 12), 67 percent (*n* = 8) maintained ART adherence ⩾80 percent for all assessments.

**Figure 1.**
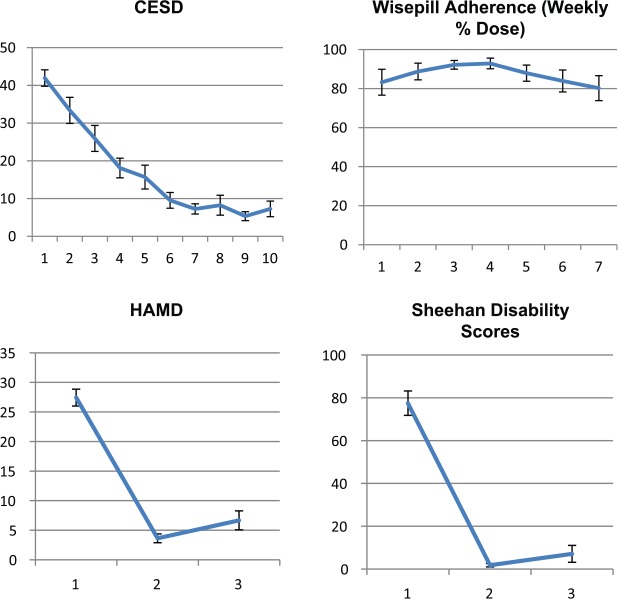
Graphs of estimated means from the generalized mixed models. Note that error bars refer to standard errors.

See Figure 1 for a depiction of results.

## Discussion

This study is a first step in adapting and evaluating CBT-AD among PLWH in South Africa using a task-sharing approach (Ziphamandla). Several formative steps were accomplished in this study. First, the Ziphamandla intervention was acceptable to patients as evidenced by patient retention. Second, the implementation of the Ziphamandla intervention was feasible as measured by patient retention and provider fidelity to intervention delivery. These findings are consistent with similar task-sharing psychotherapy implementation studies conducted in other LMICs (Rahman et al., 2008). The third formative step that was accomplished in this study was the collection of preliminary data that supports the association of the Ziphamandla intervention with improvements in depressive symptoms and functioning among PLWH. These results are consistent with studies on CBT-AD conducted elsewhere (Safren et al., 2009, 2012; Simoni et al., 2013).

Regarding adherence, of those who completed treatment (*n* = 12), 67 percent maintained optimal ART adherence (⩾80%) throughout the study period. However, we did not find significant improvements in ART adherence following treatment. Study inclusion criteria were based upon depressive symptoms, not ART non-adherence, and as such, there may have not been sufficient variability in ART non-adherence at baseline to detect a meaningful change in ART adherence over an 8-week period. Figure 1 shows that the most pronounced improvements in adherence were from baseline to week 4, immediately following the Life Steps module of Ziphamandla. However, longer follow-up data were limited as there were missing Wisepill data at the 3-month follow up due to participants not continuing to charge their Wisepill devices. This is an implementation challenge that will need to be addressed in future studies. Furthermore, although there are strengths in using Wisepill, the positive adherence effect of medication monitoring has been well established and can only be ruled out with the inclusion of a control condition. Future studies could benefit from the inclusion of criteria that selects for ART non-adherence, inclusion of biological indicators of adherence, and longer follow-up periods. If findings continue to replicate, future adaptations to this intervention may consider a greater emphasis on Life Steps in the later treatment sessions and may also consider adding booster Life Steps sessions.

Other implementation challenges were also identified that would need to be resolved to ensure successful integration of this intervention in primary HIV care in South Africa. First, this study demonstrates the challenges one may encounter when task-sharing CBT via lay counselor delivery, in particular the demands for intensive training and supervision. The degree of supervision provided in this study could not realistically be emulated in a public health clinic in South Africa. Potential strategies for decreasing supervision hours need to be considered and integrated into future research. Furthermore, despite the intensive training and supervision, the therapists’ fidelity to intervention delivery was low at times, with an average of only 54 percent of generic CBT elements and 63 percent of session-specific components being addressed across sessions. This highlights the necessity for supervisors to review session content on an ongoing basis with the therapists in supervision. However, the review of treatment sessions would require a further time commitment by the supervisors, which potential supervisors in a public health clinic may not have. These feasibility issues need to be addressed in future studies, perhaps using ongoing booster training sessions and peer supervision models.

There are a number of limitations to note in this study including small sample size and the absence of a comparison condition. The almost exclusively female sample may limit generalizability of findings to men. A further potential limitation is the slightly different recruitment procedure by clinic setting, and any implications of the differing procedures in recruiting the patient population. Also, the effects of the intervention on depressive symptoms are larger than what would have been expected from a six-to-eight session behavioral intervention. It is possible that the reliability of this effect is limited by the small sample size, and it is also difficult to interpret the size of the effect in the absence of a randomized, comparison condition. The reliability of the fidelity ratings may have also been biased by the small sample size. Furthermore, the reliability and validity of the isiXhosa translation of the CES-D could not be determined due to the small sample. Participant self-reporting of depressive symptoms over time may have also been biased by perceived investigator expectancy. Nevertheless, despite these limitations, the preliminary data promote further investigation of the efficacy of the Ziphamandla intervention in treating depression and improving adherence in PLWH in South Africa.

Given the resource constraints in primary HIV care in South Africa, the identification of an acceptable and feasible therapy model that is effective in treating depression and improving adherence and can be administered by a non-mental health specialist is imperative for the provision of mental health care in this population (Lund et al., 2010). This study is an important first step in adapting CBT techniques for adherence and depression for the South Africa setting using a task-sharing approach. Addressing depressive symptoms and non-adherence in the context of HIV care in South Africa has the potential for reducing morbidity and mortality and improving secondary HIV prevention. Further research addressing the limitations and implementation challenges of this study is needed to validate and ensure sustainable delivery of this intervention. A randomized, controlled trial of the Ziphamandla intervention in PLWH in South Africa, with a cost-effectiveness analysis, is essential.

## Appendix 1

### Description of the Ziphamandla intervention

#### Module 1. Psychoeducation and motivational interviewing

The first session of the intervention focused on educating patients on depression, psychotherapy, and the CBT model, and on enhancing patient motivation. Because depression is not a familiar concept among our population and neither is psychotherapy, it was important to help patients understand their disorder and to provide them with an understanding of the therapeutic process in general and CBT specifically.

#### Module 2. Life Steps

Given the importance of ART adherence in PLWH, the second session of the treatment was dedicated to the adherence intervention called Life Steps. Life Steps consists of 11 educational, cognitive behavioral, and problem-solving steps. The goal of the intervention is to identify structural and psychological barriers to adherence and problem-solving ways to overcome these barriers. A detailed description of Life Steps can be found elsewhere (Safren et al., 2001).

The primary adaptation made to Life Steps was the replacement of the suggested plans and back-up plans with culturally relevant suggestions. For example, for Life Step 4: Obtaining Medications, the suggestion of setting up a medication mail-in order, which is not possible in South Africa, was replaced with the suggestion of having a friend or family member pick up the medication at their local clinic on the patient’s behalf, which is possible. The order of the steps was also changed so they reflected as closely as possible the sequence of events that the patients follow, that is, transportation to the clinic, obtaining medication (at the clinic), and storing medication (when they get home from the clinic). Two additional steps were added to the Life Steps intervention: identifying social supports and planning for taking medication when using substances. A manual of the adapted Life Steps adherence intervention was created and published in South Africa (Andersen et al., 2013).

#### Module 3. Activity scheduling

The primary adaptation of the activity scheduling module was the creation of a new activity list. Activities that are common in the United States, but are not common in South Africa, were replaced by activities that are more culturally relevant (e.g. going to church, having your hair braided, going to Mzoli’s—a famous barbeque joint in the township of Gugulethu).

#### Module 4. Problem-solving

No adaptations were made to the problem-solving module (other than the translation of the worksheets into isiXhosa). The module consisted of the interventionist and the participant collaboratively identifying a problem to work on, creating a comprehensive list of possible solutions to the problem, deciding on a possible solution for the participant to attempt, and breaking the solution into manageable steps for the participant to complete. The goal of this module was to teach the skill of problem-solving across a range of problems.

#### Module 5. Relaxation training

To further simplify and standardize the treatment administration, a recording was made in isiXhosa of the progressive muscle relaxation exercise. This allowed the nurses to play the recording after demonstrating and rehearsing the diaphragmatic breathing technique with the patient. A copy of the recording was also provided to each patient for use at home.

All the worksheets used in treatment were translated into isiXhosa for the benefit of the patients. As the Ziphamandla training was conducted in English, the manual remained in English; the vocabulary, however, was adjusted when needed.
